# Correction: Remapping the foundations of morality: Well-fitting structural model of the Moral Foundations Questionnaire

**DOI:** 10.1371/journal.pone.0303714

**Published:** 2024-05-09

**Authors:** Michael Zakharin, Timothy C. Bates

In Table 1, the best-fitting model from the Harper & Rhodes (2021) and its fit metrics are incorrect. The correct best fitting model is a three-factor model instead of five and its fit metrics should have been 0.87 in CFI and 0.07 in RMSEA. Please see the correct Table 1 here.

**Table 1:** MFQ fit metrics obtained in the previous studies.

Study	N	Metrics reported		MFQ version	Best fitting model	Sample description
		RMSEA	CFI			
Curry et al. (2019)	1,042	0.050	0.910	MFQ-30	7 factors	UK online sample
Graham et al. (2011)	413	0.043	0.876	MFQ-30	5 factors	Eastern Europe online sample
Iurino & Saucier (2018)	8,055	0.084	0.853	MFQ-20	5 factors (modified)	Survey of World Views international sample
Yalçındağ et al. (2019)	1432	0.104	0.850	MFQ-30	5 factors	Three Turkish samples
Graham et al. (2011)	411	0.039	0.841	MFQ-30	5 factors	Latin America online sample
Graham et al. (2011)	299	0.042	0.838	MFQ-30	5 factors	South Asia online sample
Davies et al. (2014)	3994	0.063	0.829	MFQ-30	5 factors	New Zealand national probability sample
Graham et al. (2011)	26,014	0.048	0.824	MFQ-30	5 factors	US online sample
Graham et al. (2011)	1,670	0.046	0.811	MFQ-30	5 factors	Western Europe online sample
Kivikangas et al. (2017)	874	0.078	0.749	MFQ-30	5 factors	Finnish nationally representative sample
Ji & Janicke (2018)	234	0.077	0.744	MFQ-30	Hierarchical	Chinese students
Kim, Kang & Yun (2012)	478	0.068	0.681	MFQ-30	5 factors	South Korean students
Nilsson & Erlandsson (2015)	540	0.072	0.679	MFQ-30	5 factors	Swedish students
Ji & Janicke (2018)	204	0.078	0.658	MFQ-30	Hierarchical	US students
Harper & Rhodes (2021)	322	0.070	0.87	MFQ-30	3 factors	UK online sample
Hadarics & Kende (2017)	403	0.091	0.681	MFQ-30	5 factors	Hungarian student sample
Doğruyol et al. (2019)	4,971	0.050	0.940	REL-15	5 factors	Many Labs 2 Project, Western countries
Doğruyol et al. (2019)	1,997	0.060	0.940	REL-15	5 factors	Many Labs 2 Project, Non-Western countries
Du (2019)	761	0.080	0.930	REL-15	5 factors	Two Chinese samples
