# Supplementary material for: Remapping the foundations of morality: Well-fitting structural model of the Moral Foundations Questionnaire
Source: PLoS One. 2021 Oct 22;16(10):e0258910. doi: 10.1371/journal.pone.0258910 (PMC8535174; doi:10.1371/journal.pone.0258910)
Supplement: Attachment — Submitted filename: Response to Reviewers.docx [file pone.0258910.s001.docx]

Dear Dr. Jonason,

Thank you for giving us the opportunity to submit a revised draft of the manuscript “Remapping the foundations of morality: Well-fitting structural model of the Moral Foundations Questionnaire” for publication in the journal PLOS ONE.

We appreciate the time and effort that you and the reviewers dedicated to providing feedback on our manuscript and are grateful for the insightful comments on and valuable improvements to our paper. We have accepted and addressed all comments; below each of these is addressed point-by-point, with reviewers’ original comments are listed below (in italics) followed by our response to each comment.

We hope that the paper is now acceptable for publication in the journal.

Sincerely,

Michael

**Comments from Reviewer 1**

**Comment:** *The strength of the paper is its empirical analyses, but it requires substantial re-framing and engagement with the literature.*

**Response:** We thank the reviewer for their positive evaluation of the empirical analyses and for their direction to additional citations in the literature. We have incorporated these below.

**Comment:** *And here is where the current paper seems really out of touch with the literature, almost treating the question of the MFQ validity as an open question, when in fact, in dozens of studies, it has been shown to be invalid. So it is not true to say that “the predictive validity of the MFQ has been generally supported, especially in the domain of politics”. Rather just the opposite. What has been supported are correlations between the two, that’s about it.*

**Response:** Thank you for this suggestion. We were happy to reword this sentence removing the implication of prediction. We now say “*Since its development, the correlation of the MFQ with external measures has been widely studied, especially in the domain of politics*.” (See lines 118-119 page 5)

**Comment:** *So, let’s go back to the beginning. Kevin and I were perhaps among the first wave of people to find that the MFQ does not reliably factor into 5 dimensions in 10 populations (Smith et al- which btw your paper cites incorrectly), but at best 2, the structure differed by country (US and AU). This paper was not a critique and in fact we had no interest in finding a different factor structure. I suggest a reread of it.*

**Response:** We apologise for not noting that Smith et al. (2017) and Hatemi et al. (2019) used multiple samples. Also, for using the Smith et al. (2017) reference to cover both Smith et al. (2017) and Hatemi et al. (2019) work. We have corrected the reference and now say (see line 136-140, pages 5-6)

“*Smith et al. and Hatemi et al. [25, 26], using multiple samples, reported that the MFQ did not reliably factor into 5 dimensions, but rather into 2, with structure differing between the US and Australia. They also found that moral foundations are not stable across time and that MFQ scores reflect rather than cause political attitudes. Smith et al. [26] also reported that the MFQ foundations show no evidence of heritability*”

**Comment:** *Then came Iurino and Saucier and many others who hit this more definitely (see Harper and Rhodes and Davis et al who find MFT is not valid across non US/white populations, and just the other day Hadarics Márton’s paper, then of course, the heterogeneity in MFTs within and across ideologies identified by Frimer; then finding that measure is not stable across time (2 years= Smith et al), and then the findings that MFT appears to be caused by, rather than cause, the traits it is proposed to influence (for attitudes or political orientation see Hatemi Crabtree and Smith, Kivikangas et al, Ciuk, Everett et al., Strupp-Levitsky et al (Jost), and Márton and Kende among many others . Finally it’s not heritable (Smith et al.).*

**Response:** Thank you for this helpful comment: We now cite each of these papers mentioned in the revision. We say (See lines 141-151 page 6 and lines 185-191, page 9):

“*Strupp-Levitsky et al. [27] suggest that rather than being causal, the foundations build on other, more basic, variables such as empathy, need for closure and need for cognition. In a study manipulating partisan and group identity cues by embedding these in modified MFQ items, Ciuk [28] reported that item endorsement was affected by partisan alignment, supporting the conclusion that causality runs from political ideology to moral foundations. At the psychometric level, Iurino and Saucier [5] tested measurement invariance of the 5-factor structure of the MFQ in 27 countries and concluded that there was little support for a five-factor solution for the questionnaire. Similarly, in a US sample, Davis et al. [29] tested measurement invariance of the MFQ in Black and White samples, concluding that the assumption of scalar invariance could not be supported. Jointly, these pose considerable challenges for a measure that aims to be culturally universal, perhaps especially problems in finding a well-fitting model as a basis for prediction*.”

We continue

“*More recently, Harper & Rhodes [32] tested the factor structure of the MFQ in two British samples (total N = 750), confirming that the proposed five-factor structure was not psychometrically sound according to accepted metrics. They also tested an extended MFQ, including the nine items of the sixth “Liberty” foundation proposed by Haidt and colleagues [12]. Adding the Liberty scale, however, did not lead to a well-fitting six-factor model, and instead was better explained by a three-factor model comprising “traditionalism”, “compassion” and “liberty*”.

**Comment:** *In this, I think the paper needs to redo the front end. The above papers are not critiques, so it a bit disingenuous to frame them as such. Rather, most of them were studying a trait of interest, and in so doing found out the predictor variable (MFQ) was garbage*.

**Response:** We have clarified that these papers use the MFQ as a trait of interest. We say “*moral foundations theory and the MFQ measure have been criticized both on theoretical and empirical grounds (e.g. by papers using it as a trait of interest*)”. See line 127-129, page 5.

**Comment:** *The comparison of fits in the paper seem highly selective.*

**Response:** We are not quite sure what this comment refers to. We added two new references to Table 1 (see page 8), citing Harper & Rhodes (2021) and Hadarics & Kende (2017) findings, both supporting the conclusion that MFQ falls short of the acceptable degree of model fit. In the text we now say “*No reports, to our knowledge, have resulted in satisfactory fit (see Table 1 for a sample of fits for different MFQ models in different samples and cultures).”,* lines 168-170, page 7.


**Comment: …***One cannot simply just restructure the MFQ into different domains, without then updating the theory. …The MFQ is the proposed measure of Haidt MFT theory. It has specific logic, organizing principles evolutionary roots etc that link the domains to each other and the MFQ questions. [The present] paper is centered around offering a different factor structure to the data that conflicts with the theory as written. What are you competing hypotheses? What parts of your findings invalidate Haidt’s theory? What part support it? What parts of Haidt’s theory require modification based on your results, what part require abandonment?*

*This is important because if one wanted to use this new formulation as a means to lets say, run different analyses, like behavior genetic ones for example, then this should be the paper to address those questions. Otherwise it is simply a data exercise – which is not a bad thing, but just limited in what it offers.*

**Response:** We agree that enumerating which parts of our findings invalidate Haidt’s theory and which support it, and, thus, which parts of Haidt’s theory require modification based on our results is important, and we strove in the discussion to do this. We have taken a second look at the discussion rewriting where possible to make the differences and similarities between the MFQ predictions and our seven-factor model clearer. Regarding the individualizing foundations we now say on lines 416-425, page 18:

*“The harm/care and fairness/reciprocity foundations reproduced with perfect fidelity: that is for each of these foundations, all 6 items loaded on a single factor in the 7-factor model, supporting the MFT.”*

Regarding three binding foundations we say:

“*By contrast, the well-fitting model draws firm distinctions between sanctity and purity and between loyalty to country and loyalty* *to what we termed clan (combining family and community*)”.

We have also added a figure showing the mapping from the 5 hyphenated original foundations to the 7-factor model. (Figure 6).

This suggests a need to modify MFT to account for additional distinct evolutionary selection pressures which would cause distinct sanctity and purity systems (rather than a single system processing both these kinds of information) and distinct systems for loyalty to kin and to country (rather than a single system processing both these kinds of information).

In this work we were constrained by the existing MFQ items which limits our ability to propose and test competing hypotheses. We did not enter with competing hypotheses, but rather generated these in interpreting the results of study 1, before replicating them in four independent datasets. We plan to test our model in future work and regarding this we now say (on lines 609-612, page 26):

“*A future theory of moral judgment will need to propose and test hypotheses about the selective pressures on loyalty to country vs loyalty to kin and purity vs sanctity factors.* *In addition, constructing a modified measure of moral foundations may require generating new items to cover the larger number of domains*”

**Comment:** *One important and serious concern. You need permissions to use other peoples replication data for anything other than replication. Posting data for replication is for replication. Here you are using replication data for novel purposes. Since you used my data and I was never asked, I’m suspecting you did the same for others. It is an ethical question to take replication data in the manner you’re using it.*

**Response:** Thank you for noting this. We have emailed the corresponding authors informing them about our use of their data in our work, receiving positive replies from all of them. We note in the text the licensing of each of these open access datasets (Studies 2-5) covered by licenses allowing any use of data, not only replication, as follows (see lines 766-775, pages 32-33):

Study 2: CC0 license (You can copy, modify, distribute and perform the work, even for commercial purposes, all without asking permission)

<https://dataverse.harvard.edu/dataset.xhtml?persistentId=doi:10.7910/DVN/SJTRBI>

Study 3: CC0 license

https://dataverse.harvard.edu/dataset.xhtml?persistentId=doi:10.7910/DVN/WTUGFZ

Study 4: CC BY 3.0 license (Free to remix, transform, and build upon the material for non-profit purpose)

https://data.mendeley.com/datasets/sbmwmsynxk/1

Study 5: CC BY 4.0 license (Free to remix, transform, and build upon the material for any purpose)

<https://frontiersin.figshare.com/articles/dataset/Data_Sheet_1_The_Association_Between_Disgust_Sensitivity_and_Negative_Attitudes_Toward_Homosexuality_The_Mediating_Role_of_Moral_Foundations_xls/8234243>

**Comments from Reviewer 2**

**Comment:** *This is one of the empirically strongest papers on the Moral Foundations Questionnaire, with strengths that the paper itself accurately touts. But there are some caveats and weaknesses of which the authors seem unaware.*

**Response:** We thank the reviewer for this fulsome complement.

**Comment:** *The samples are – except for Study 5, the smallest sample – based on predominantly Western respondents (in fact, predominantly Anglo-American). Page 16 claims that study 1 identified a ‘reliable basic structure’ but this might be true only for certain populations. In general, the paper needs more caveats about potential inapplicability to non-Western populations. I don’t think we should be having a few Chinese university attendees representing (standing in for) the entire non-Western world as is done here.*

**Response:** We agree that the lack of global and cultural coverage is a limitation of the study. We now mention this in the Limitations section of the paper’s discussion saying ‘*though, given that we used only one non-Western sample, this requires validation*” See line 709, page 30

**Comment:** *With respect to the one non-Western sample, the paper fails to note important details (the sample was evidently almost 80% female) and there are some questions. What universities were the respondents from and how Westernized were they? Why was this particular sample (among all non-Western samples administered the MFQ) chosen, and did that involve cherry-picking the sample most likely to be supportive of the select model?*

**Response:** Thank you for pointing this out. In addition to stating that the sample is 80% female in the participants’ section, we now note this in the Limitations section of the paper’s discussion. We say “*This sample also had higher proportion of females (78 %) compared to other samples*”. See lines 717-718, pages 30-31. We used this sample because it was the only non-western sample with MFQ data available to us.

**Comment:** *One could also wonder if there is some degree of cherry-picked subjects in studies 1-4. The description of the study 1 sample does not rule out that these are all moral-psychology enthusiasts who volunteered. The study 2 was highly motivated – found their way to a website on moral psychology. Study 3 and 4 samples were from MTurk, but might be called ‘cream of the crop’ MTurkers based on the selection criteria; that’s well and good unless it means these results are dependent on using ‘elite questionnaire-response professionals’ and are ungeneralizable to a typical population. What percentage of MTurk participants meet the criteria specified, and were any MTurk participants recruited but then eliminated (in order to make favorable results more likely)? (A general take-home message might be that the intended structure of the MFQ is fragile, hard to locate in your typical noisy data.)*

**Response:** We agree that this is a potential limitation of the study. We have added this as a limitation. We now say in the Limitations section of the paper’s discussion (lines 721-725, page 31:

*“It is also worth mentioning that in Study 2 we used data provided by participants who voluntarily visited a moral psychology website and, presumably, were highly motivated. Similarly, in Study 3-4 we used data provided only by MTurk participants with high approval rate. This may have affected how representative of general population these samples are”.*

**Comment:** *The models employed are entered in a particular order that was apparently set a priori (is there a pre-registration to verify this?), but the question is raised whether, when all is said and done, one (or more) of them might be unnecessary. That is, perhaps by backward removal of the element adding least, a more parsimonious outcome might be reached. This is analogous to the forward versus backward methods in stepwise regression. Related questions: How does it make sense to enter a hierarchical model (which includes the five-factor model) before one enters the five-factor model, and what is the difference between ‘two-factor model’ and ‘binding and individualizing’ foundations in, for example, Table 8.*

**Response:** Thank you for this suggestion about parsimony and reducing a five-factor model rather than adding to it. Reducing the five-factor model to smaller number of factors made model’s fit worse, not better, we also added a sentence about our approach, which is “*Our approach was to develop models increasing in complexity from the simplest predicted model, to more complex structures, as required to achieve good fit*”. See page 11, lines 248-250 We did not, unfortunately pre-register this method.

**Comment:** *Following that same thread: The conclusion (though unstated) would seem to be that one can use the MFQ, but must really analyze it or make sense of its scores in a complicated and onerous manner.*

**Response:** We include a scoring system at item level and sharing the models which can be used to generate scores, available at OSF page of the paper. However, we agree that it would desirable to add items to balance the new scales.

**Comment:** *That is perhaps a problem for construct validity. An important area of future implications would be this: What do these results suggest about how* *to construct a better moral ‘foundations’ measure (e.g., one that would not be so profoundly affected by method variance, or by the pull of an underlying two-factor model and of political leanings and of acquiescence or general morality vs. amorality? In other words, how can the measurement of moral foundations be cleaned up in these regards?*

**Response:** Thank you for this suggestion, we now include very similar wording in the modelling implications section (See lines 691-693, pages 29-30 and lines 609-612, page 26):

“*Constructing a better measure of moral foundations may also require rephrasing of some items and perhaps creating new ones to remove or at least minimize the effects of method variance and possible bias*”

and

“*A future theory of moral judgment will need to propose and test hypotheses about the selective pressures on loyalty to country vs loyalty to kin and purity vs sanctity factors. In addition, constructing a modified measure of moral foundations may require generating new items to cover the larger number of domains*.”.

Smaller matters:
**Comment 7:** *The middle paragraph on page 26 leads to considerable head-scratching. The first sentence is incomprehensible. As for the 2nd, why is social dominance picked out so prominently among all possible alternatives? What is the ‘alignment’ referred to there? It would seem that one interpretation of the results is that ‘tilt’ and binding-individualizing make independent contributions because one cannot reduce the latter entirely to tilt (as some treatments of the topic have implied in the past), there being both liberal and conservative ways of endorsing binding AND of endorsing individualizing morality.*

**Response:** Thank you for this correction. We removed first sentence and provided a reference for the possible link between the tilt and social dominance. We say:

“*Future study could usefully focus on understanding this factor, including relating it to other constructs, for instance one or other component of the social dominance construct [48], which was previously offered as an explanation of liberal-conservative differences [56]*”. See lines 642-645, page 28.

**Comment*:*** *In Table 8 it is noteworthy that moving from model 3 to model 4 yielded a huge improvement in fit, which was not the case in other samples. Does this say something about how Chinese respondents characteristically handle morality or this questionnaire?*

**Response:** Thank you for pointing this out. The improvement from model 3 to model 4 (adding Judgment and Relevance method factors) was present in all samples. One explanation why it was more pronounced in the Chinese sample could be the way self-reflection (relevance items) and evaluative judgments (judgment items) are presented in the Chinese language, however since it was the only non-western sample we decided not to speculate about the cause of this effect. We mentioned this anomaly in the discussion, saying about this sample “*It also showed the largest model fit improvement by modelling two method factors*” see lines 718-719, page 31.

**Comment:** *Page 13 identifies a set of three MFQ items and labels them as sanctity, but it is hard to apply this interpretation to the third item mentioned (about roles for men and women). One could just as easily label this factor as ‘traditional gender roles/expectations’ (that also would fit 2 of the 3 items).*

**Response:** We agree with the reviewer that the alternative name for this factor is possible, however, we chose to keep the term sanctity as this is the usage from Haidt, in which both gender roles and religion are sanctified, e.g. “If the body is a temple housing divinity within, then people should not be free to use their bodies in any way they please; rather, moral regulations should help people to control themselves and avoid sin and spiritual pollution in matters related to sexuality, food, and religious law more generally” (Haidt and Graham, 2007).

**Comment:** *Page 13-14 separate out a patriotism factor from a loyalty factor, but it would seem more informative to label one of them as loyalty to country and the other as loyalty to family/team/group (i.e., to smaller-scope entities).*

**Response:** Thank you for this suggestion. We have re-worked the discussion for study 1 and ongoing discussion to pay close attention to clarity in terminology and adopted a clearer naming scheme. We say (see lines 420-424, page 18):

*“This seven (rather than five) foundation model broke-out items from the sanctity/purity, authority/respect, and ingroup/loyalty foundations to form independent sanctity and purity foundations, and independent foundations of loyalty to country and loyalty to clan. These changes also altered the nature of the authority foundation, leaving it more obviously aligned around hierarchy.”*

See lines 420-424, page 18. We have also added a figure showing the mapping from the 5 hyphenated original foundations to the 7-factor model. (Figure 6).

**Comment:** *In reference to same on page 24, it is implied that Herbert Simon differentiated patriotism from loyalty, but this seems unlikely given how Simon’s viewpoint is stated. Clarity needed.*

**Response:** Thank you for pointing this out. We have clarified that this refers to hierarchy. We say (see lines 599-605, page 26)

*“This distinction of loyalty to one’s group and obedience to hierarchy, has a distinguished history in theory. In his work on administrative behavior, Simon [54] identified loyalty and obedience as the two necessary conditions for the existence of organisations, defining loyalty as the capacity to introject organizational objectives in place of one’s own aims and obedience as choosing to make one’s default response be to follow requests of a superior: a definition which corresponds closely to notions of respecting the wishes of those in authority.”*

We also call this factor (consisting of 4 Authority items) as “hierarchy” throughout in the paper.

**Comment:** *Until the very last part of the paper, there is a tendency to label one latent variable -- created by constraining all items to load positively on it – as General Morality although an equally plausible interpretation is Acquiescence. Would be better to mention both possible interpretations from the beginning.*
**Response:** Thank you for the suggestion. We now call this general/acquiescence factor throughout in the paper.

**Comment:** *In Table 1, the “(modified)” notation for the best-fitting model for the Lurino and Saucier (2018) paper needs some explanation.*

**Response:** Thank you for pointing this out. We now explain “modified” in the table’s footnote. We say: “*Iurino & Saucier (2018) used alternative model derived from exploratory factor analysis of original MFQ items*”. See Table 1, page 8,

**Comment:** *The tendency to overestimate one’s personality traits is (on p. 12) mischaracterized as halo effect. Halo effect is more commonly used to refer to overestimation of someone else’s positive traits. Doing it to yourself is self-enhancement or social desirability bias.*
**Response:** Thank you for this correction. We now describe these as “self-enhancement or social desirability”. See line 302-303, page 13.

**Comment:** *It seems that some text on pages 17-18 is redundant with what was said before in the paper, this needs a check.*

**Response:** Thank you for pointing this out. We removed the redundant text.
